# Bony nasolacrimal duct size and outcomes of nasolacrimal silicone intubation for incomplete primary acquired nasolacrimal duct obstruction

**DOI:** 10.1371/journal.pone.0266040

**Published:** 2022-03-28

**Authors:** Min Kyu Yang, Ho-Seok Sa, Namju Kim, Jeong Hun Kim, Hokyung Choung, Sang In Khwarg

**Affiliations:** 1 Department of Ophthalmology, Asan Medical Center, University of Ulsan College of Medicine, Seoul, Korea; 2 Department of Ophthalmology, Seoul National University Bundang Hospital, Seoul National University College of Medicine, Seongnam, Korea; 3 Department of Ophthalmology, Seoul National University Hospital, Seoul National University College of Medicine, Seoul, Korea; 4 Department of Ophthalmology, Seoul Metropolitan Government-Seoul National University Boramae Medical Center, Seoul, Korea; Cairo University Kasr Alainy Faculty of Medicine, EGYPT

## Abstract

**Purpose:**

To investigate the association between the bony nasolacrimal duct (NLD) size and outcomes of nasolacrimal silicone intubation for incomplete primary acquired nasolacrimal duct obstruction (PANDO).

**Methods:**

Patients who underwent silicone intubation for incomplete PANDO and had undergone facial computed tomography (CT) were included. Surgical success was judged by both epiphora improvement and normalized tear meniscus height (TMH; < 300 μm) on anterior segment optical coherence tomography at 3 months after tube removal. The area, major axis diameter, and minor axis diameter of the elliptic bony NLD sections were measured in 1.0 mm-thick axial CT images. These bony NLD sizes were analyzed for associations with surgical success and TMH normalization.

**Results:**

Eighty-one eyes of 48 patients were investigated. The smallest area and the smallest minor axis diameter were significantly larger in the success group (49 eyes), compared with those in the failure group (median smallest minor axis diameter: 4.7 mm vs. 3.8 mm, P = 0.008, Mann–Whitney U test). There was also a tendency for the TMH normalization rate to significantly increase as the smallest area and the smallest minor axis diameter increased (P = 0.028 and 0.037, respectively, Fisher’s 2 × 4 tests). Under multivariable logistic regression analysis using generalized estimating equation, a larger smallest minor axis diameter was associated with success of the nasolacrimal silicone intubation (odds ratio: 2.481, 95% confidence interval: 1.143–5.384).

**Conclusion:**

Surgical success of the nasolacrimal silicone intubation in incomplete PANDO is associated with a larger smallest minor axis diameter of the bony NLD. This finding will help understand the pathophysiology of surgical failure after nasolacrimal silicone intubation.

## Introduction

Primary acquired nasolacrimal duct obstruction (PANDO) is characterized by chronic inflammation of the nasolacrimal duct (NLD) without any definite cause [1]. Numerous factors, such as sinusitis and exposure to topical antiglaucoma drugs, have been proposed as etiologic factors for the development of PANDO. Bony NLD morphology has also been suggested as an etiologic factor of PANDO, but its role is still controversial [2].

The traditional treatment for complete PANDO is dacryocystorhinostomy (DCR). For incomplete PANDO, surgical options such as balloon dacryoplasty and nasolacrimal silicone intubation have been applied, achieving results comparable with DCR [3, 4]. Nasolacrimal silicone intubation preserves the NLD structure and facilitates natural lacrimal drainage [4]. Some studies have investigated the factors associated with the failure of nasolacrimal silicone intubation in incomplete PANDO [4–6]. However, there have been no studies involving morphometric evaluation of the bony NLD and the outcomes of nasolacrimal silicone intubation.

In this study, we investigate the association between the size of the axial bony NLD section and the surgical outcomes of nasolacrimal silicone intubation in incomplete PANDO.

## Methods

Patients who underwent nasolacrimal silicone intubation for incomplete PANDO at Seoul National University Hospital and Seoul National University Bundang Hospital from January 2008 to December 2019 and also had undergone a 1.0 mm thickness axial facial CT scan were searched via a retrospective review of the electronic medical records. Patients with punctal or canalicular stenosis, dacryocystitis, history of facial bone fracture, facial nerve palsy, and eyelid malposition, such as entropion and ectropion, were excluded. Patients younger than 40 years of age were also excluded in consideration of progressive bony NLD enlargement until 40 years old [7, 8]. The study was conducted according to the tenets of the Declaration of Helsinki, and the study protocol was approved by the institutional review boards of Seoul National University Bundang Hospital. Informed consent was not given, as patient records and informations were anonymized and de-identified prior to analysis.

**Fig 1:**
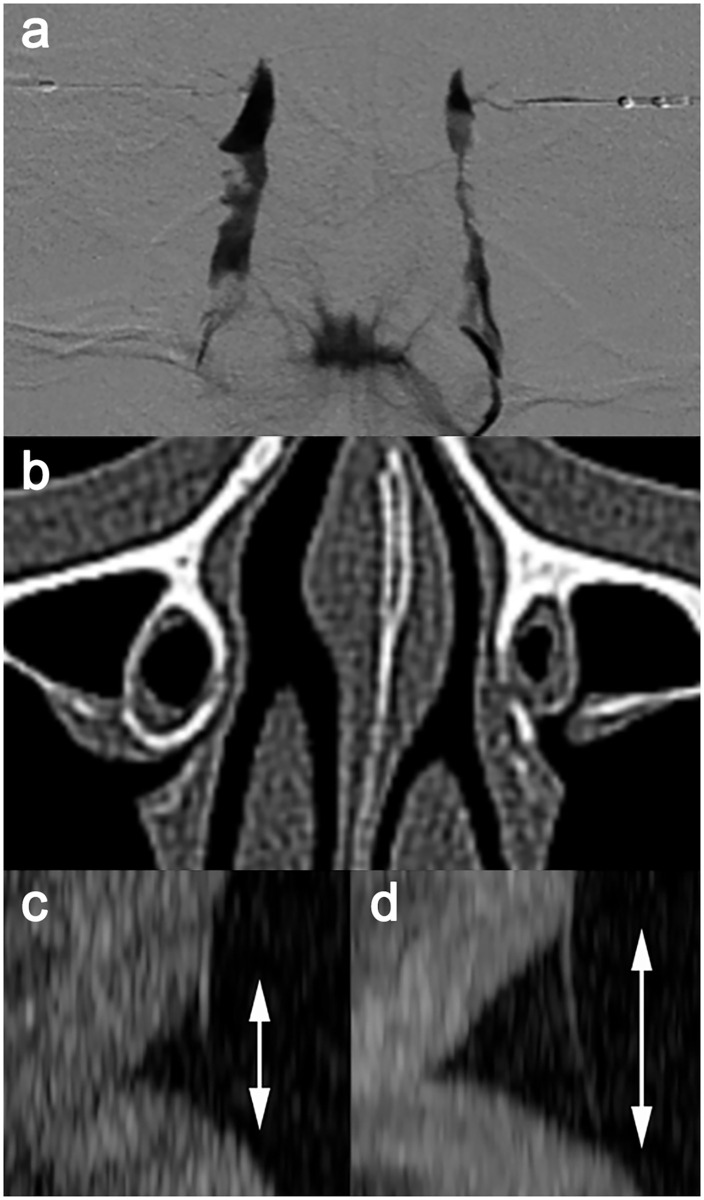
Measuring the bony nasolacrimal duct (NLD) size of a patient showed unilateral success after bilateral nasolacrimal silicone intubation. (a) Preoperative dacryocystography shows a dilated NLD in the right eye and stenosis of the sac-NLD junction in the left eye. (b) A preoperative computed tomography axial image shows that the smallest minor axis diameter of the right eye was larger than that of the left eye (white lines, 5.6 mm vs. 4.2 mm). (c, d) Anterior segment optical coherence tomography shows tear meniscus between the cornea and lower eyelid; tear meniscus height (white arrow) is normal in the right eye (298 μm) and high in the left eye (428 μm) at 8 months after surgery.

At the initial visit, patients with epiphora were evaluated by slit-lamp examination, lacrimal irrigation, and anterior segment optical coherence tomography (ASOCT). Spectralis optical coherence tomography (Heidelberg Engineering GmbH, Heidelberg, Germany) was performed using the anterior segment module, which enabled the precise measurement of tear meniscus height (TMH) [9, 10]. To avoid the effect of transient hydrodynamic dilation of the lacrimal drainage system, ASOCT was performed at least 1 h after lacrimal irrigation. Incomplete PANDO was defined as subjective epiphora (Munk score ≥ 2) [11], an increased TMH (≥ 300 μm) [9, 10, 12–14], and passage with minor amount of reflux (less than half) on lacrimal irrigation. Preoperative dacryocystography (DCG) was routinely performed (Fig 1a).

All surgical procedures were performed by two oculoplastic surgeons (NK and SIK) with the same protocol. We used Bowman lacrimal probes (Bausch + Lomb Storz Ophthalmic Instruments, St Louis, Mo, USA) and a silicone bicanalicular tube (canaliculus intubation set 8590450; Beaver-Visitec International, Inc., Waltham, MA, USA). To maximize the dilation effect, sequential dilation was performed with the largest size probe, starting from size #00 [15]. Follow-up examinations were scheduled for 1 week, 1 month, 3 months, and 6 months after intubation. A slit-lamp examination and ASOCT were performed at each visit. The silicone tube was usually removed at 3 months after intubation.

Surgical outcomes were judged as successful when two conditions were achieved and maintained until 3 months after tube removal: epiphora improvement (decrease of the Munk score to 0 or 1) and normal TMH (< 300 μm) on ASOCT. All eyes were divided into two groups according to surgical outcomes (success group and failure group). In the case of unilateral success after bilateral nasolacrimal silicone intubation, each eye was sorted separately.

CT scans were taken for various head and neck problems within 5 years before or after silicone intubation. A Brilliance 64-slice scanner (Philips Healthcare, Cleveland, OH, USA) was used for the CT scans. Scans produced non-contrast, continuous sections with acquisition parameters of 1.0 mm slice thickness, 120 kV tube voltage, and 200 mAs tube current. We chose five contiguous axial sections, including the grossly narrowest bony NLD. Because the axial section of the NLD was shaped as an ellipse, the major axis diameter was defined as the longest diameter, and the minor axis diameter was the longest line segment perpendicular to the major axis (Fig 1b). After sharpening the images with the bone window setting, the area, major axis diameter, and minor axis diameter of the bony NLD were measured per section with the embedded tools of the DICOM viewer (INFINITT PACS, INFINITT Healthcare Co., Ltd., Seoul, Korea), by a single examiner (MKY). The three smallest values from the five sections and their mean value were adopted for further analysis.

Clinical data including symptom duration, exposure to topical antiglaucoma drugs, history of sinusitis, and TMH were collected. Based on DCG, the preoperative NLD stenosis was assessed as follows: 1) extent: diffuse stenosis (length of stenosis > 2 mm) vs. focal stenosis (at 1–4 points) [16] and 2) degree: severe (faintly visible NLD) vs. weak vs. dilated. The clinical characteristics and bony NLD size parameters of the success and failure groups were compared with Fisher’s exact tests and Mann–Whitney U tests using MedCalc version 9.6.4.0 (MedCalc Software, Mariakerke, Belgium). For patients with unilateral success after bilateral surgery, bony NLD size parameters were compared with Wilcoxon tests conducted between successful and failed eyes. Additionally, all eyes were divided into four subgroups of equal width according to the smallest bony NLD size parameters. The rates of TMH normalization at 3 months after tube removal were calculated for each subgroup. Fisher’s 2 × 4 test was applied to compare normalization rates between the four subgroups. Univariable and multivariable logistic regression analyses were also performed for factors of surgical success after nasolacrimal silicone intubation. The generalized estimating equation with compound symmetry covariance matrix was applied to adjust for inter-eye correlation. The area under the receiver operating characteristic curve (AUROC) and the cut-off value for surgical outcome prediction were calculated. A P value < 0.05 was considered statistically significant.

## Results

Eighty one eyes of 48 patients met the inclusion criteria and were investigated in this study, including 22 eyes from 11 patients with unilateral success after bilateral surgery, 44 eyes from 22 patients with bilateral success or failure after bilateral surgery, and 15 eyes from 15 patients with unilateral surgery. Forty-nine eyes of 34 patients were classified to the surgical success group and 32 eyes of 25 patients were classified to the surgical failure group (success rate: 60.5%). Surgical failures were determined at 6.1 (interquartile range [IQR] 5.1–7.2) months after surgery. The patients in the surgical success group were followed-up for 7.3 (IQR 4.2–35.3) months, during which there was no recurrence of epiphora with the normal TMH. The demographics and clinical characteristics of each group are shown in Table 1. All clinical characteristics were not significantly different between the success and failure groups (median TMH: 433 μm vs. 449 μm, P = 0.516, Mann–Whitney U test). The extent and degree of NLD stenosis were similar in both groups (severe stenosis: 24.5% vs, 28.1%, P = 0.797, Fisher’s exact test).

**Table 1:** Demographics and clinical characteristics of the success and failure groups after nasolacrimal silicone intubation.

	Total	Success group	Failure group	P value^b^
	(n = 81 eyes, 48 patients)	(n = 49 eyes, 34 patients)^a^	(n = 32 eyes, 25 patients)^a^	
Male sex, No. (%)	28 (34.6)	16 (32.7)	12 (37.5)	> 0.99
Age at surgery, median (IQR), years	60 (55–68)	60 (55–66)	59 (54–70)	0.813
Symptom duration, median (IQR), months	24 (6–60)	18 (6–57)	30 (6–63)	0.280
Exposure to topical antiglaucoma eye drops, No. (%)	2 (2.5)	1 (2.0)	1 (3.1)	> 0.99
History of sinusitis, No. (%)	9 (11.1)	6 (12.2)	3 (9.4)	> 0.99
Preoperative tear meniscus height, median (IQR), μm	440 (381–489)	433 (381–483)	449 (381–497)	0.516
Preoperative nasolacrimal duct stenosis, No (%)				
Extent (diffuse vs. focal)				
Diffuse	29 (35.8)	19 (38.8)	10 (31.3)	0.636
Degree (severe vs. weak vs. dilated)				
Severe	21 (25.9)	12 (24.5)	9 (28.1)	0.797
Dilated	15 (16.0)	9 (18.4)	4 (12.5)	0.551

IQR, interquartile range.

^a^Including 11 patients with unilateral success after bilateral surgery.

^b^P values derived from Mann–Whitney U test in continuous variables and Fisher’s exact test in categorical variables.

The smallest area was significantly larger in the success group compared with that in the failure group (median: 26 mm^2^ vs. 18 mm^2^, P = 0.013, Mann–Whitney U test) (Table 2). The second and third smallest area and mean of the three smallest areas were also significantly larger in the success group (all P < 0.03). Similarly, the smallest minor axis diameter was significantly larger in the success group compared with that in the failure group (median: 4.7 mm vs. 3.9 mm, P = 0.008). The second and third smallest minor axis diameter and mean of the three smallest minor axis diameters were also significantly larger in the success group (all P < 0.02). However, none of the four values of the major axis diameter were significantly different between the success and failure groups (all P > 0.05).

**Table 2:** Comparison of the axial bony nasolacrimal duct size parameters between the success and failure groups after nasolacrimal silicone intubation.

Axial bony NLD size parameters	Success group (n = 49 eyes)	Failure group (n = 32 eyes)	P value
Area, median (IQR), mm^2^			
Smallest values	26 (21–33)	18 (15–27)	0.013
Second smallest values	27 (22–34)	20 (27–16)	0.012
Third smallest values	28 (24–36)	21 (17–27)	0.024
Mean of the three smallest values	27 (22–34)	20 (16–27)	0.012
Major axis diameter, median (IQR), mm			
Smallest values	6.4 (5.9–7.2)	5.9 (5.1–6.5)	0.068
Second smallest values	6.8 (6.1–7.5)	6.1 (5.3–6.8)	0.067
Third smallest values	7.1 (6.2–7.8)	6.3 (5.7–7.0)	0.114
Mean of the three smallest values	6.8 (6.1–7.6)	6.1 (5.4–6.6)	0.072
Minor axis diameter, median (IQR), mm			
Smallest values	4.7 (4.1–5.2)	3.9 (3.5–4.7)	0.008
Second smallest values	4.8 (4.2–5.4)	4.0 (3.6–4.9)	0.014
Third smallest values	4.8 (4.3–5.6)	4.1 (3.7–5.0)	0.013
Mean of the three smallest values	4.7 (4.2–5.3)	3.9 (3.6–4.8)	0.008

IQR, interquartile range; NLD, nasolacrimal duct.

Analysis of the patients with unilateral success after bilateral surgery revealed similar results. All four smallest values of area and minor axis diameter were significantly larger in the successful eyes than in the failed eyes (all P < 0.04, Wilcoxon test), whereas all four smallest values of the major axis diameter did not significantly differ between the successful and failed eyes (all P > 0.07). Fig 1 shows a representative case of unilateral success after bilateral nasolacrimal silicone intubation for incomplete PANDO.

**Fig 2:**
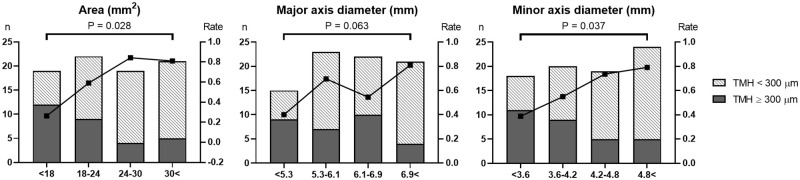
Distributions and rates of tear meniscus height (TMH) normalization (< 300 μm) at 3 months after tube removal according to the smallest bony nasolacrimal duct parameters. The P values were derived from Fisher’s 2 × 4 tests.

TMH normalization occurred in 51 eyes at 3 months after the tube removal. As the smallest bony NLD parameters increased, the TMH normalization rates increased (Fig 2). The TMH normalization rates were significantly different among the subgroups of the smallest area and the smallest minor axis diameter (P = 0.028 and 0.037, respectively, Fisher’s 2 × 4 tests).

Table 3 shows the results of logistic regression analysis using generalized estimating equation. Multivariable logistic regression analysis revealed that only the smallest minor axis diameter (odds ratio 2.481, 95% confidence interval 1.143–5.384, P = 0.022) was independently associated with surgical success after nasolacrimal silicone intubation. The prediction model with smallest minor axis diameter for surgical success had an AUROC of 0.728 (95% confidence interval: 0.605–0.836). The cut-off value of the smallest minor axis diameter for surgical success was 3.9 mm.

**Table 3:** Logistic regression analysis using generalized estimating equation for surgical success after nasolacrimal silicone intubation.

Variables	Odds ratio (95% CI)	P value
Univariable analysis		
Male sex	1.021 (0.328–3.179)	0.971
Age at surgery	0.990 (0.948–1.034)	0.647
Exposures to topical antiglaucoma eye drops	0.641 (0.379–1.084)	0.097
History of sinusitis	1.203 (0.177–8.182)	0.850
Preoperative tear meniscus height	0.999 (0.995–1.003)	0.475
Preoperative nasolacrimal duct stenosis		
Diffuse extent (vs. focal)	1.323 (0.399–4.384)	0.647
Severe degree (vs. weak or dilated)	0.763 (0.210–2.768)	0.681
Smallest major axis diameter	1.779 (1.008–3.138)	0.047
Smallest minor axis diameter	2.839 (1.434–5.620)	0.003
Multivariable analysis		
Smallest minor axis diameter	2.481 (1.143–5.384)	0.022

CI, confidence interval.

## Discussion

In this study, we found that a larger size of the axial bony NLD section was associated with better surgical outcomes of the nasolacrimal silicone intubation in incomplete PANDO. Previous studies aimed to identify factors associated with the success of nasolacrimal silicone intubation; canalicular obstruction, younger age, and good passage without resistance or regurgitation were reported as good prognostic factors [4–6]. We analyzed our results while controlling for these confounding factors to clearly document the impact of bony NLD size. Only patients with no canalicular stenosis and minor amount of reflux on lacrimal irrigation were included in the present study. The mean age and the degree of preoperative NLD stenosis did not significantly differ between the success and failure groups.

Janssen et al. [17] reported the result of balloon dacryoplasty and the smallest diameter of the bony NLD in incomplete PANDO patients. The study had a similarity with our study in the use of silicone intubation; in that, balloon dacryoplasty also enhances natural lacrimal drainage through the NLD. In this previous study, the smallest diameter of the bony NLD was larger in the success group than in the failure group (median: 3.4 mm vs. 2.8 mm), though not by a significant amount. However, a similar comparison in our study showed a statistically significant difference. It may be due to the larger size of our patient population and more precise measurement of bony NLD parameters with a 1.0 mm slice thickness CT.

A histopathologic study revealed chronic inflammation of the NLD mucosa in PANDO patients [1]. Although the pathological changes of NLD mucosa after silicone intubation have not been clearly elucidated, several studies have suggested that an NLD mucosal inflammatory reaction to silicone material may occur. This reaction can lead to progressive fibrosis, granuloma, or granulation tissue formation [18–20], eventually resulting in recurrent obstruction of the NLD [21]. We attempted to maximally expand the NLD mucosal lumen using a sequential dilatation technique and to suppress inflammatory swelling of the NLD mucosa by topical steroids [22], but a smaller bony NLD may limit the mucosal luminal expansion. The narrow NLD lumen increases flow resistance and tear stagnation that enhance the vicious inflammatory cycle in the NLD mucosa [22]. Further studies measuring the mucosal luminal diameter of NLD on postoperative CT will be helpful to validate our hypothesis.

The bony NLD morphology as a causative factor for PANDO is controversial. Ali and Paulsen [2] concluded that the current general consensus in the literature is that the bony NLD diameter is not a significant factor in the etiology of PANDO. However, even if previous studies showed no significant difference in bony NLD diameter between PANDO patients and control group, it cannot be definitively concluded that this anatomical factor has no effect, as the duration of chronic inflammation of NLD can be different among subjects. Even with a large bony NLD diameter, PANDO may eventually develop if inflammation of the NLD persists for a long time. All previous studies have evaluated the association of the bony NLD diameter and PANDO without considering the duration of inflammation. Since we determined the surgical failure after a similar follow-up period in this study, our results will have less bias caused by the differences in the duration of inflammation.

We included patients with bilateral success or failure (45.8% of total patients). If the conditions of both eyes were symmetric and included together in the analysis, it may bias the result of analysis. However, both NLDs were completely separated and their sizes were not identical in any patient. Exposure to topical antiglaucoma eye drops and sinusitis involvement were unilateral in all patients of our study. Moreover, patients with unilateral success after bilateral surgery accounted for a significant portion (22.9% of total patients). In all of these patients, bony NLD size parameters were also larger in successful eyes than in failed eyes, with statistical significance. Therefore, including patients with bilateral success or failure in analysis is not expected to produce erroneous results.

In the present study, the size of the bony NLD section was directly measured from CT axial images. Several studies have shown that the narrowest part is located in the middle and upper portion of the bony NLD [23–25]. However, the section with the smallest anteroposterior diameter and the section with the smallest transverse diameter do not always coincide [23, 25]. In a Japanese cadaver study, the section with the smallest anteroposterior diameter lies 3.6 ± 2.3 mm from the entrance of the bony NLD, which was 2.0 mm closer in comparison with the section with the smallest transverse diameter [25]. Hence, we chose five contiguous axial sections, including the grossly narrowest section of the bony NLD, to cover a 5.0 mm length of the bony NLD.

Unlike a narrow NLD, other known risk factors of PANDO, such as exposure to topical antiglaucoma eye drops and a history of sinusitis, were not associated with surgical failure in our study. The effects of topical antiglaucoma eye drops and sinusitis might be less than those in studies dealing with PANDO development, because postoperative NLD inflammation was suppressed with tobramycin/dexamethasone eye drops, and the postoperative follow-up period was relatively shorter than the duration of PANDO development.

There were several limitations in our study. First, the study had a retrospective design. Second, the diameter of the bony NLD does not strictly reflect the actual diameter of the NLD. The diameter of the actual NLD lumen can be variable if the mucosal lining of the NLD is swollen due to inflammation. Third, only Koreans were included in our study. Indeed, the mean diameters of our groups overlapped with the results of prior studies on Asian populations, and differed from results found for Caucasians [8, 17, 23, 26]. A further prospective study that includes a large number of patients is needed to support our results and to set the criteria for prediction of surgical outcomes. Performing CT before silicone intubation is not a common practice, and radiation effects and cost of CT should also be considered. However, the use of cone-beam CT-DCG has the advantage of reducing radiation exposure, and Papathanassiou et al. [27] have argued that it should be used as a diagnostic standard for PANDO, instead of the conventional DCG. If a patient is reluctant to undergo DCR or has poor general condition, CT scans can be helpful to make an evidence-based decision making with ease for the patients to understand.

In conclusion, our study revealed that the surgical success of nasolacrimal silicone intubation is associated with a larger smallest minor axis diameter of the bony NLD sections in incomplete PANDO. This finding aids the understanding of the pathophysiology of surgical failure after nasolacrimal silicone intubation.
